# Pneumococcal colonization among tracheostomy tube dependent children

**DOI:** 10.1371/journal.pone.0206305

**Published:** 2018-10-19

**Authors:** Guliz Erdem, Anirudh K. Singh, Anthony J. Brusnahan, Amber N. Moore, William J. Barson, Amy Leber, Jorge E. Vidal, Serkan Atici, Samantha J. King

**Affiliations:** 1 College of Medicine, The Ohio State University, and Nationwide Children’s Hospital, Columbus, Ohio, United States of America; 2 Center for Microbial Pathogenesis, Nationwide Children’s Hospital, Columbus, Ohio, United States of America; 3 Department of Pathology and Laboratory Medicine, Nationwide Children’s Hospital, Columbus, Ohio, United States of America; 4 Hubert Department of Global Health, Rollins School of Public Health, Emory University, Atlanta, Georgia, United States of America; 5 Department of Pediatrics, Marmara University School of Medicine, Istanbul, Turkey; Oregon Health & Science University, UNITED STATES

## Abstract

*Streptococcus pneumoniae* colonization is a precursor to pneumococcal disease. Although children with a tracheostomy have an increased risk of pneumococcal pneumonia, the pneumococci colonizing their lower airways remain largely uncharacterized. We sought to compare lower respiratory tract isolates colonizing tracheostomy patients and a convenience sample of isolates from individuals intubated for acute conditions. We collected pneumococcal isolates from the lower respiratory tract of 27 patients with a tracheostomy and 42 patients intubated for acute conditions. We compared the penicillin susceptibility, rates of co-colonization, genetic background, and serotype of isolates colonizing these patient populations. Isolates from both groups showed high genetic diversity. Forty multi-locus sequence types and 20 serotypes were identified. There was no significant difference in serotype distribution, co-colonization rates, vaccine coverage, or non-susceptibility to penicillin among pneumococcal isolates from the two groups. Colonization of the lower airways with non-vaccine serotypes 15B/C, 23B and 35B was noted for the first time in patients with tracheostomies and supports recently observed increases in nasopharyngeal colonization and disease due to these serotypes.

## Introduction

Asymptomatic colonization of the nasopharynx is considered a precursor for clinical pneumococcal disease. Levels of colonization are higher in children than adults and certain groups of patients could be at increased risk of developing pneumococcal infection [1–3]. Patients with tracheostomies have pneumococcal colonization of their lower respiratory tract; however, little is known about the strains colonizing these patients [4, 5]. A study published in 1979 examined pneumococcal colonization of 27 children (mean age of 8.5 years) biweekly over a one year period [5]. Pneumococci were isolated from 22.5% of the 444 samples taken; although, 22 of those isolates were from patients with active pneumonia. A more recent study of tracheostomy patients, both adults and children, at a long-term care facility revealed 17% of cultures were positive for pneumococci [4]. This study also defined the serotypes of endotracheal isolates. They did not differentiate the isolates from adult and pediatric patients, but the serotypes of the isolates were 23F, 19F, 17F, 15A, 6A and some non-typeable.

*Streptococcus pneumoniae* is a diverse species, as demonstrated by the >90 serotypes [6]. While the 7-valent conjugate vaccine (PCV7) reduced invasive disease and colonization by vaccine types, some other serotypes increased in prevalence [7, 8]. Introduction of PCV13 further reduced pneumococcal disease, but recent reports indicate similar increases in colonization and disease due to some non-vaccine serotypes [9, 10, 11].

Although strains have different propensities to cause disease, studying strains colonizing patients with underlying conditions may identify pneumococcal lineages likely to cause disease. Isolates colonizing paediatric patients with tracheostomies have rarely been evaluated. Thus, we compared the serotypes, susceptibility to penicillin, and genetic background of pneumococci colonizing the lower respiratory tract of children with tracheostomies and a convenience sample of children intubated due to acute non-pneumococcal illness.

## Materials and methods

Medical records at Nationwide Children’s Hospital, Columbus, Ohio from January 2013 to December 2014 were reviewed following Institutional Review Board (IRB) approval (IRB16-00812). Given that the study was reviewed as Risk Level 1, no greater than minimal risk, and involved retrospective chart review, the IRB waived the requirement for informed consent. We identified tracheostomy patients from which a pneumococcal isolate was recovered from deep tracheal aspirates. Deep tracheal aspirates were collected by trained medical staff and respiratory therapists, using sterile technique and standard airway precautions, thus minimizing the risk of contamination with upper respiratory tract flora to the extent possible. A single isolate from each patient was used. If the same patient was hospitalized multiple times during the study period, the first pneumococcal isolate obtained was used in the study. No individual had documented pneumococcal disease within the prior year. This resulted in 27 isolates for use in the study. For comparison we used a convenience sample of the 42 isolates from deep tracheal aspirates patients intubated for acute conditions and cultured within 24 hours of admission obtained over the same study period. The majority of patients were children; however, but a few older than 18 years of age were also treated at our hospital due to their pre-existing chronic conditions. Whether there were any significant differences between the patient populations were assessed using a two-tailed chi-squared test with Yate’s correction (P ≤ 0.05). The exception was for age, where statistical significance of differences was assessed by a two-tailed Student’s *t* test.

Samples were cultured using conventional media including sheep blood, chocolate and MacConkey agars with and plates incubated at 37°C in 5% CO_2_. Pneumococci were identified by optochin susceptibility, latex agglutination or Vitek GP Cards (bioMerieux). Antimicrobial susceptibilities were performed using Etest strips (bioMerieux) with established breakpoints [12]. Isolates were recovered from frozen stocks and grown for DNA preparation using previously described methodology [13]. Multi-locus Sequence Typing (MLST) was used to define the genetic background of isolates (http://pubmlst.org/spneumoniae/). Genetic relatedness and clonal complexes, named for the predicted founder, were inferred using goeBURST available in PHYLOViZ 2.0. Novel alleles are available in GenBank (accession numbers MG675061-MG675064) and added to the pneumococcal MLST database.

Serotypes were assigned using multiplex PCR [14]. The serotype of multiplex-negative isolates was determined by the Quellung reaction (Statens Serum Institute). Non-typeable isolates were screened for genes that replace the capsule locus in some cases, *pspK* [15], *aliC* (*aliC*F AGATGCCAAATGGTTCACGC and *aliC*R GCGCTTTGTTATACCTAGATGTTTC) and *ali*D [16]. A two-tailed chi-squared test with Yate’s correction was used to identify any significant differences (P ≤ 0.05) between the two groups of isolates.

## Results

All tracheostomy patients had underlying conditions associated with respiratory failure requiring chronic ventilation including Trisomy 18, DiGeorge syndrome, muscular dystrophy, traumatic injuries with spinal cord transection, severe cerebral palsy, congenital tracheal abnormalities and cystic lymphangiomas (S1 Table). The convenience sample included 15 previously healthy children admitted due to viral bronchiolitis, burns, acute onset seizures, drowning or complications during elective surgical procedures including tonsillectomy and ear ventilation tube placement. Nineteen patients had chronic conditions that included cerebral palsy, chronic seizure disorders, hypoxic ischemic encephalopathy, congenital hydronephrosis, obstructive sleep apnea, cartilage hair dysplasia, bronchiectasis, laryngeal cleft, chromosomal abnormalities, eosinophilic esophagitis, heart transplant, brain stem glioma, Stevens Johnson syndrome, pulmonary nodules and asthma (S2 Table). These patients did not have tracheostomy tubes. In addition, eight children had tracheomalacia/laryngomalacia, cough and prematurity.

Due to the retrospective nature of the study the vaccination status of some children is unknown; however, of the children old enough to be vaccinated against pneumococcus 77.7% of the tracheostomy group and 89.5% of the convenience sample were reported as such (Table 1 and S1 and S2 Tables). The median age of the patients was lower in the convenience sample (2 years, interquartile range 0.9–7) compared with the tracheostomy group (5 years, interquartile range 2–9). One of the patients in the tracheostomy group and three of the patients in the control group were immunocompromised (S1 and S2 Tables). There were no statistically significant differences between the patient population in vaccination status, age, sex, immune status, recent antibiotic use and hospitalization (Table 1).

**Table 1:** Patient characteristics.

	Tracheostomy tube patients (n = 27)	Convenience sample (n = 42)	P value^2^
Median Age (interquartile range)	5 (2–9)	2 (0.9–7)	0.99
Male	14 (51.9%)	29 (69.0%)	0.24
Immunocompromised	1 (3.7%)	3 (7.1%)	0.95
Antibiotic use last 2 weeks	1 (3.7%)	1 (2.4%)	0.75
Hospitalization	15 (55.6%)	26 (61.9%)	0.78
Reported pneumococcal vaccination	21 (77.7%)	34^1^ (86.8%)	0.37

^1^ N = 38 as we excluded 4 patients under 2 months as it is below the recommended age for pneumococcal vaccination.

^2^ Calculated by chi-squared with Yate’s correction with the exception of age for which the means were compared using a two-tailed Student’s *t* test.

Co-isolation of other potentially pathogenic bacterial species was not significantly different between the groups (63.0% of tracheostomy vs 42.9% of convenience sample isolates P = 0.17) (Tables 2 and 3). Viruses detected are reported (S1 and S2 Tables), but as it is difficult in many cases to be certain whether these viruses are the cause of the encounter diagnosis or co-colonizing we did not compare the rates between groups. All immunocompromised patients were co-colonized, with other bacterial species (S1 and S2 Tables). The distribution of non-susceptibility to penicillin was not significantly different between the groups (resistant and intermediate, 33.3% of tracheostomy vs 38.1% of convenience sample isolates, P = 0.88).

**Table 2:** Details of pneumococcal isolates.

Isolate	Serotype	Sequence Type^3^	Clonal Complex^4^	Penicillin MIC^5^	Bacterial Co-colonization^6^
**Isolates from tracheostomy tube patients**					
1	19F	654	CC654	0.03	-
2	35B	1204	CC558	0.5(I)	*M*.*c*
3	11A/D	62	CC63	0.01	*E*., *S*.*a*., *S*.*m*.
4	21	432	CC432	0.01	*P*.*a*.
5	15 B/C	1757	CC199	0.01	*K*.*p*., *S*.*a*., *S*.*m*.
6	28A	546	CC546	0.09	*P*.*a*., *S*.*a*.
7	15 B/C	199	CC199	0.04	-
8	22F/A	433	CC433	0.01	*M*.*c*., *P*.*a*.
9	21	432	CC432	0.02	*H*.*i*., MRSA
10	35B	558	CC558	0.75(I)	-
11	15 B/C	199	CC199	<0.01	*M*.*c*.
12	15 B/C	**11360**	CC1262	0.04	*C*., *P*.*a*.
13	23B	439	CC439	0.04	-
14	11A/D	62	CC63	0.03	*E*., *H*.*i*.
15	23A	**12425**	CC156	0.25(I)	*P*.*a*, *S*.
16	10A	816	CC460	0.03	-
17	NT^1^	2315	CC2315	0.19(I)	-
18	15A/F	63	CC63	0.25(I)	*A*. *M*.*c*. *P*., *P*.*a*., *S*.*d*.
19	9V/A	**13967**	CC15	0.04	*P*.*a*., *S*.
20	35B	558	CC558	1.5(I)	*P*.*a*.
21	23B	1373	CC156	0.09	-
22	19F	1203	CC346	2(R)	-
23	35B	3632	CC558	1.5(I)	*E*., *M*.*c*.
24	22F/A	7314	CC433	0.04	-
25	35B	558	CC558	0.38(I)	*K*.*p*., *S*.
26	6C/D	8446	CC156	0.04	MSSA
27	19F	425	CC395	0.03	-
**Convenience sample**					
1	11A/D	62	CC63	0.12(I)	-
2	11A/D	62	CC63	0.01	-
3	35B	10493	CC558	2(R)	-
4	6 C/D	473	CC473	0.04	-
5	15 B/C	199	CC199	0.02	*M*.*c*.
6	35F/47F	1635	CC460	0.06	-
7	19F	320	CC320	4(R)	-
8	15 B/C	**12061**	CC156	0.5(I)	-
9	3	13499	CC180	0.03	-
10	19F	654	CC654	0.12(I)	*P*.*a*., *R*.
11	19F	654	CC654	0.06	MSSA
12	15 B/C	3280	CC156	0.19(I)	*M*.*c*.
13	6C/D	473	CC473	0.03	-
14	3	13499	CC180	0.06	-
15	19A	1451	CC320	4(R)	*M*.*c*.
16	15 B/C	199	CC199	0.04	*M*.*c*.
17	19A	8748	CC156	0.09	*M*.*c*., MSSA
18	23B	439	CC439	0.03	-
19	16F	659	CC659/965	0.03	-
20	11A/D	62	CC63	0.04	-
21	22F/A	433	CC433	0.04	-
22	19F	654	CC654	0.04	*H*.*i*, *M*.*c*.
23	6C/D	**11413**	CC1390	0.04	*N*.*m*., MSSA.
24	16F	10439	CC383	0.04	-
25	38/25F/25A	393	CC393	0.04	*H*.*i*.
26	23F	36	CC439	0.06	*M*.*c*.
27	11A/D	62	CC63	0.03	-
28	23B	1373	CC156	0.12(I)	MSSA
29	16F	659	CC659/965	0.01	-
30	6C/D	1379	CC1379	0.38(I)	*H*.*i*.
31	21	432	CC432	0.04	-
32	NT^2^	**13966**	CC15	0.25(I)	-
33	23B	439	CC439	0.04	*H*.*i*.
34	6C/D	473	CC473	0.5(I)	*M*.*c*.
35	11A/D	62	CC63	0.04	MSSA
36	35B	558	CC558	1.5(I)	-
37	19A	1451	CC320	3(R)	-
38	22F/A	433	CC433	0.06	*M*.*c*.
39	19A	1451	CC320	3(R)	-
40	23A	338	CC156	0.5(I)	*H*.*i*. MSSA
41	23A	338	CC156	0.19(I)	-
42	24 F/A/B	162	CC156	0.04	-

^1^NT *cspA* and *pspK* negative and *aliD* and *aliC* positive.

^2^*cspA*, *pspK*, *aliC* and *aliD* negative confirmed by quelling.

^3^Bold text indicates sequence types novel to this study.

^4^Defined as a group of STs where each strain is a single locus variant of at least one other strain in the group.

^5^Minimum inhibitory concentration; I indicates intermediate levels of penicillin susceptibility and R non-susceptibility to penicillin (9). All other strains are susceptible to penicillin.

^6^Abbreviations for co-colonizing bacterial species: *A*., *Acinetobacter* of unspecified species; *C*. *Corynebacterium* of unspecified species; *E*., *Enterobacter* of unspecified species; *H*.*i*., *Haemophilius influenzae*; *K*.*p*., *Klebsiella pneumonia*; MRSA, methicillin resistant *Staphylococcus aureus*; *M*.*c*., *Moraxella catarrhalis*; *N*.*m*., *Neisseria meningitidis*; *P*., *Proteus* of unspecified species; *Ps*., *Pseudomonas* of unspecified species; *P*.*a*., *Pseudomonas aeruginosa*; *R*., *Rothia* of unspecified species; *S*., *Serratia* of unspecified species; *St*., *Staphylococcus* of unspecified species; *S*.*a*., *Staphylococcus aureus*; *S*.*m*. *Stenotrophomanas maltophila*; Group G *Streptococcus*.

**Table 3:** Data summary.

	Strains from tracheostomy tube patients	Strains from convenience sample	P value^1^
Bacterial co-colonization	63.0%	42.9%	0.17
Non-susceptibility to penicillin	33.3%	38.1%	0.88
PCV13 coverage	14.8%	26.2%	0.41
Serotypes reported increased post PCV13 (23B, 15 B/C, 35B)	40.7%	21.4%	0.15

^1^ Calculated by chi-squared with Yate’s correction.

MLST demonstrated high diversity, but did not support a clonal lineage for strains colonizing children with a tracheostomy. Only 13 of the 40 STs identified represented multiple isolates and six were new STs. The most common STs were ST62 (N = 7), ST199 (N = 4), ST558 (N = 4) and ST654 (N = 4). The 40 STs were members of 23 clonal complexes. The most common of which, CC156 (N = 10), CC63 (N = 8) CC558 (N = 7) and CC199 (N = 5), represented both patient populations.

The 69 strains included 20 serogroups/serotypes. Most of the frequently identified serotypes were common in both groups. The majority of isolates were serotypes not included in PCV13 (85.2% of tracheostomy vs 73.8% of convenience sample isolates, P = 0.41). Although pneumococcal vaccination was not specifically documented, the majority of patients were reported as appropriately vaccinated (77.7% of tracheostomy vs 89.5% of convenience sample P = 0.37). One isolate from each group was non-typeable. One these isolates encoded AliC and AliD [16]; while no alternative capsular locus genes were identified in the other.

## Discussion

We observed no differences in rates of co-colonization with other potentially pathogenic bacteria between the two groups. We don’t believe this was influenced by the distribution of immunocompromised patients. As only 3.7% of the tracheostomy patients and 7.1% of the convenience sample were immunocompromised. A wide range of different co-colonizing bacteria were observed. As co-colonization rates with any specific species or genus were low it was not possible to investigate differences in distribution of individual species between the tracheostomy and control groups.

We observed no significant differences between the isolates from tracheostomy patients and the convenience sample for susceptibility to penicillin or vaccine coverage (Table 3). This observation is important as no studies following introduction of PCV13 have studied pneumococci colonizing the lower airway of paediatric tracheostomy patients. In fact, there have been very few studies of pneumococcal colonization of pediatric patients with a tracheostomy tube. We cannot compare our study with that of Brook *et al*. 1979 as our study was aimed at examining the isolates from tracheostomy patients and not the rates of colonization [5]. The study by Adler *et al*. 2012 identified the serotype of isolates; however, they did not differentiate the isolates from adult and pediatric patients [4]. The serotypes of the 45 isolates from the trachea or trachea and nasopharynx were 23F, 19F, 17F, 15A, 6A and non-typeable. The limited numbers of serotypes in this study are likely for two reasons. Firstly, patients were sampled between one and three times and it is not clear how many of the isolates were from the same patients and therefore potentially the result of the same colonization event. Secondly, strains were likely spread between patients at this long-term care facility. In contrast, patients included in our study were only at Nationwide Children’s for relatively short periods of time prior to sampling. Variation in the serotype distribution as compared to those in our study may reflect differences in vaccination status, which is not discussed, or geographical and temporal patterns of colonization [4].

**Fig 1:**
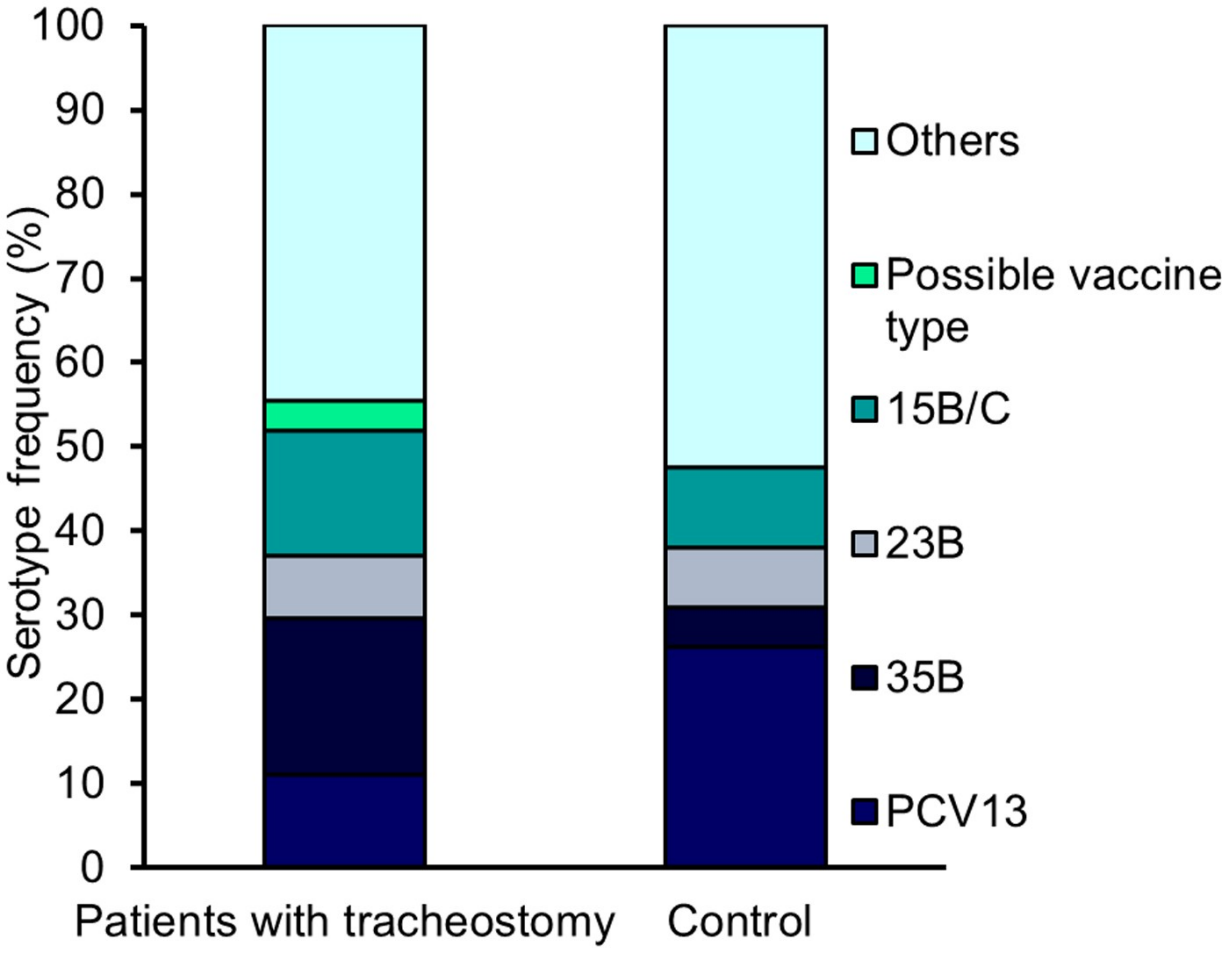
Frequency of emerging serotypes within the tracheostomy and control patient populations. The percentage of strains in both patient populations representing serotypes in PCV13, emerging serotypes 15B/C, 23B and 35B, and non-vaccine types is reported. The PCR typing scheme cannot distinguish 9V and 9A. PCV13 includes 9V, but not 9A, hence it is unclear if the isolate classified in this group is a vaccine type and therefore it is reported as a possible vaccine type.

As colonization precedes disease, the serotypes observed can predict those likely to be increasingly observed in disease. Colonization of the lower airways with the non-vaccine serotypes 15B/C, 23B and 35B was noted for the first time. Previous studies have reported post-PCV13 increases in colonization due to non-vaccine serotypes including 23B, 15B/C and 35B [9, 17, 18]. Our data suggest higher numbers of these serotypes in the tracheostomy patients; although the difference is not significant with the available sample size (40.7% vs 21.4%, Fig 1, P = 0.15).

The increase in colonization and disease due to 35B has been attributed to increased presence of ST588 [10, 18]. Our data also suggest that there is an increase in isolation of single locus variants of ST588 post-PCV13. A previous study suggested ST199 that expressed the 19A capsule and was prevalent in the PCV7 era is now associated with serogroup 15 [10]. Our isolates included four ST199 isolates all of which were 15B/C. The pathogenic potential of these isolates is not known, but these data suggest there should be further monitoring of the distribution of these isolates in colonization and disease.

The retrospective nature of the study leads to some limitations including the lack of nasopharyngeal samples, the fact that strains were only isolated at a single time point and that for some patients we did not have confirmed vaccination records. However, this work adds to the current understanding of pneumococci colonizing the airway of children with a tracheostomy. The number of children with a tracheostomy, while increasing, is relatively small and it is possible some of the differences observed in this study would be statistically significant if there were a larger sample size. Despite this, colonization of the lower airways with non-vaccine serotypes 15B/C, 23B and 35B was noted for the first time in patients with tracheostomies and supports recently observed increases in nasopharyngeal colonization and disease due to these serotypes.

## Supporting information

S1 Table Clinical information for the patients with a tracheostomy. (DOCX)

S2 Table Clinical information for the convenience sample. (DOCX)
