# Supplementary material for: Pneumococcal colonization among tracheostomy tube dependent children
Source: PLoS One. 2018 Oct 19;13(10):e0206305. doi: 10.1371/journal.pone.0206305 (PMC6195293; doi:10.1371/journal.pone.0206305)
Supplement: S1 Table — (DOCX) [file pone.0206305.s001.docx]

**S1 Table. Clinical information for the patients with a tracheostomy.**

| **Patient** | **Age^#^**  **(year)** | **Sex** | **Encounter diagnosis** | **Chronic condition*** | **Admission to hospital** | **PICU admission** | **Antibiotic use within the last two weeks** | **Pneumococcal**  **Vaccination** | **Viral pathogens detected** |
| --- | --- | --- | --- | --- | --- | --- | --- | --- | --- |
| 1 | 6 | F | Bronchiolitis | Spondylometaphyseal dysplasia | Yes | No | No | Yes | RSV |
| 2 | 1.3 | F | Cardiac surgery | Di George syndrome, tetralogy of Fallot | Yes | Yes | No | Yes | No |
| 3 | 10 | F | Pain, increased size of cystic hygroma | Cystic hygroma | Yes | No | No | Yes | No |
| 4 | 2 | M | Cough, runny nose | Tracheobronchomalacia, subglottic stenosis | No | No | No | Yes | No |
| 5 | 2 | M | Cough, runny nose | Pierre-Robin sequence, ventriculoperitoneal shunt, hemophagocytic lymphohistiocytosis* | No | No | No | Yes | No |
| 6 | 3 | M | Fever, runny nose | Cystic hygroma | No | No | No | Yes | No |
| 7 | 5 | F | Surveillance culture | Hypoxic ischemic encephalopathy, adrenal insufficiency | No | No | No | Yes | No |
| 8 | 4 | F | Surveillance culture | Spinal cord injury with quadriplegia | No | No | No | Yes | No |
| 9 | 2 | F | Surveillance culture | Fetal hypokinesia | No | No | No | Yes | No |
| 10 | 9 | F | Abdominal surgery | Hypertrophic cardiomyopathy, tracheoesophageal fistula | Yes | No | No | Yes | No |
| 11 | 7 | F | Chronic secretions | Prematurity with complications of developmental delay, tracheomalacia | No | No | No | Yes | No |
| 12 | 12 | F | Surveillance culture | Trisomy 18 | No | No | No | Yes | No |
| 13 | 10 | F | Feeding tube placement | Muscular dystrophy, scoliosis, restrictive lung disease | Yes | No | Yes, clindamycin | Unknown | No |
| 14 | 5 | F | Seizure | Bronchopulmonary dysplasia, cerebral palsy | Yes | Yes | No | Yes | No |
| 15 | 3 | M | Fever, viral respiratory tract infection | Tracheal ring | No | No | No | Yes | No |
| 16 | 4 | M | Desaturation | Acrofacial dysostosis | Yes | No | No | Yes | Metapneumovirus |
| 17 | 5 | F | Bronchoscopy | Agenesis of the left lung, intraventricular hemorrhage | Yes | No | No | Yes | No |
| 18 | 22 | M | Seizure | Cerebral palsy, quadriplegia | Yes | Yes | No | Yes | Rhinovirus |
| 19 | 11 | M | Surveillance culture | Subdural empyema, cerebellar herniation, cerebellar stroke, muscle spasticity | No | No | No | Unknown | No |
| 20 | 15 | F | Bronchiolitis with respiratory failure | Cerebral palsy | Yes | Yes | No | Yes | Rhinovirus/enterovirus |
| 21 | 0.6 | M | Viral bronchiolitis | Trisomy 21, congenital hypothyroidism, tracheomalacia, severe obstructive sleep apnea | Yes | No | No | Yes | Parainfluenza |
| 22 | 3 | M | Bronchiolitis | Spinal cord transection | Yes | No | No | Yes | Influenza, rhinovirus |
| 23 | 0.25 | M | Cerebral hemorrhage, status epilepticus | Hypoxic ischemic encephalopathy, tetralogy of Fallot | Yes | Yes | No | Unknown | No |
| 24 | 2 | M | Tonsillectomy | Trisomy 7, obstructive sleep apnea | Yes | Yes | No | Unknown | No |
| 25 | 15 | M | Surveillance culture | Cerebral palsy, vocal cord paralysis | No | No | No | Unknown | No |
| 26 | 7 | M | Bleeding from tracheal aspirate | Trisomy 18 | Yes | No | No | Yes | No |
| 27 | 8 | M | Bronchoscopy | Tracheal stenosis | No | Yes | No | Unknown | No |

Abbreviations/Descriptions:

^#^: Patients younger than two years of age are reported as a fraction of year.

*: Immunocompromised

PICU: Pediatric intensive care unit

RSV: Respiratory syncytial virus

Rhinovirus/enterovirus: The PCR method used in these cases does not differentiate between these two viruses.
