# Supplementary material for: Pneumococcal colonization among tracheostomy tube dependent children
Source: PLoS One. 2018 Oct 19;13(10):e0206305. doi: 10.1371/journal.pone.0206305 (PMC6195293; doi:10.1371/journal.pone.0206305)
Supplement: S2 Table — (DOCX) [file pone.0206305.s002.docx]

**S2 Table. Clinical information for the convenience sample.**

| **Patient** | **Age^#^**  **(year)** | **Sex** | **Diagnosis** | **Chronic or preexisting condition*** | **Admission to hospital** | **PICU admission** | **Antimicrobial use**  **within the last**  **two weeks** | **Pneumococcal Vaccination** | **Viral pathogens detected** |
| --- | --- | --- | --- | --- | --- | --- | --- | --- | --- |
| 1 | 0.04 | M | Bronchiolitis with respiratory failure | No | Yes | Yes | No | N/A | RSV |
| 2 | 0.09 | F | Bronchiolitis with respiratory failure | No | Yes | Yes | No | N/A | RSV |
| 3 | 7 | M | Traumatic brain injury | No | Yes | Yes | No | Yes | No |
| 4 | 0.08 | M | Bronchiolitis with respiratory failure | No | Yes | Yes | No | N/A | RSV |
| 5 | 1 | M | Bronchiolitis with respiratory failure | No | Yes | Yes | Yes, amoxicillin | Yes | RSV, rhinovirus |
| 6 | 2 | F | Croup with respiratory failure | No | Yes | Yes | No | Yes | Parainfluenza |
| 7 | 6 | M | Bleeding to airway after tonsillectomy | Obesity, obstructive sleep apnea | Yes | Yes | No | Yes | No |
| 8 | 1.08 | F | Bronchiolitis | Asthma | Yes | Yes | No | Yes | RSV |
| 9 | 12 | F | Respiratory failure, bronchiolitis | Congenital hydronephrosis, Tourette syndrome | Yes | Yes | No | Yes | No |
| 10 | 14 | M | Respiratory distress | Brainstem glioma* | Yes, deceased | Yes | No | Yes | No |
| 11 | 12 | M | Severe burn with respiratory failure | No | Yes | Yes | No | Unknown | No |
| 12 | 1.9 | M | Chronic cough | Tracheomalacia | No | Yes | No | Yes | Rhinovirus |
| 13 | 0.03 | M | Bronchiolitis | No | No | Yes | No | No | RSV |
| 14 | 20 | M | Aspiration pneumonia | Cerebral palsy, hydrocephalus | Yes | Yes | No | Yes | No |
| 15 | 2 | F | Dysphagia | Laryngeal cleft | No | Yes | No | No | No |
| 16 | 2 | F | Bronchiolitis with respiratory failure | No | Yes | Yes | No | Yes | No |
| 17 | 2 | M | Drowning | No | Yes, deceased | Yes | No | Yes |  |
| 18 | 1.9 | F | Status epilepticus, bronchiolitis | Epilepsy | Yes | Yes | No | Yes | Parainfluenza |
| 19 | 20 | M | Altered mental status, respiratory failure | Hypoxic ischemic encephalopathy | Yes | Yes | No | Yes | Rhinovirus/Enterovirus |
| 20 | 20 | M | Status epilepticus, resp. failure | Lennox-Gestaut syndrome | Yes | Yes | No | No | No |
| 21 | 1.9 | M | Evaluation for chronic cough | Tracheomalacia | No | No | No | Yes | No |
| 22 | 8 | F | Severe burn, respiratory failure | No | Yes | Yes | No | Yes | No |
| 23 | 10 | M | Pulmonary nodules | Pulmonary nodules | No | No | No | Yes | No |
| 24 | 0.04 | M | Apnea, bronchiolitis | Prematurity | Yes | Yes | No | N/A | RSV |
| 25 | 8 | F | Bronchiectasis | Cartilage hair dysplasia, ciliary dyskinesia* | Yes | Yes | No | Yes | No |
| 26 | 0.4 | F | Dysphagia | Cough | No | No | No | No | Rhinovirus, adenovirus |
| 27 | 7 | M | Evaluation for wheezing | Bronchomalacia, asthma | No | No | No | Yes | No |
| 28 | 4 | M | Severe burn, respiratory failure | No | Yes | Yes | No | Yes | No |
| 29 | 0.8 | M | Evaluation for stridor | Stridor, eosinophilic esophagitis | No | No | No | Yes | No |
| 30 | 6 | M | Status asthmaticus, bronchiolitis | Asthma, bronchomalacia | Yes | Yes | No | Yes | RSV |
| 31 | 3 | F | Asthma attack | Asthma | Yes | Yes | No | Yes | No |
| 32 | 2 | M | Evaluation for stridor, tonsillectomy | Laryngomalacia | No | No | No | Yes | No |
| 33 | 8 | M | Cough | Stevens Johnson syndrome due to Mycoplasma infection | No | No | No | Yes | No |
| 34 | 3 | M | G-tube leak and low grade fever with cough | Heart transplant* | No | Yes | No | Yes | No |
| 35 | 4 | M | Severe burn with respiratory failure | No | Yes | Yes | No | Yes | No |
| 36 | 11 | M | Lung resection for gastropulmonic fistula | Cavitary lung lesion | No | Yes | No | Yes | No |
| 37 | 0.9 | M | Cough, and wheezing | Cough | No | Yes | No | Yes | No |
| 38 | 0.4 | M | Severe burn | No | Yes | Yes | No | Yes | No |
| 39 | 1.5 | F | Respiratory distress, bronchiolitis | Trisomy 21, congenital heart disease, subglottic stenosis | Yes | Yes | No | Yes | Adenovirus, coronavirus, parainfluenza |
| 40 | 1.08 | F | Aspiration pneumonia | Cough | No | Yes | No | Yes | No |
| 41 | 4 | M | Cough | Cough | No | No | No | Yes | No |
| 42 | 1.6 | M | Bronchiolitis | No | No | Yes | No | Yes | RSV |

Abbreviations/Descriptions:

^#^Patients younger than two years of age are reported as a fraction of year.

*Immunocompromised

N/A: Not applicable

PICU: Pediatric intensive care unit

RSV: Respiratory syncytial virus

Rhinovirus/enterovirus: The PCR method used in these cases does not differentiate between these two viruses

Severe burn: Inhalation injury, burn to vital areas or burn >25% of body surface area reported
